# Analysis of antibody profiles in symptomatic malaria in three sentinel sites of Ivory Coast by using multiplex, fluorescent, magnetic, bead-based serological assay (MAGPIX™)

**DOI:** 10.1186/s12936-015-1043-2

**Published:** 2015-12-21

**Authors:** David Koffi, André Offianan Touré, Marie-Louise Varela, Inès Vigan-Womas, Sylvain Béourou, Somela Brou, Marie-France Ehouman, Laeticia Gnamien, Vincent Richard, Joseph Allico Djaman, Ronald Perraut

**Affiliations:** Unité de Paludologie, Institut Pasteur de Côte d’Ivoire, Abidjan, Côte d’Ivoire; Unité d’Immunologie, Institut Pasteur de Dakar, Dakar, Sénégal; Unité d’Immunologie des Maladies Infectieuses, Institut Pasteur de Madagascar, Antananarivo, Madagascar; UFR Biosciences, Université Félix Houphouet Boigny, Abidjan, Côte d’Ivoire; Unité d’Epidémiologie, Institut Pasteur de Dakar, Dakar, Sénégal

**Keywords:** Malaria, *Plasmodium falciparum*, ELISA, IgG, Surface antigens, Multiplex, MAGPIX, Ivory Coast, Symptomatic malaria, Biomarkers

## Abstract

**Background:**

Advances in malaria control have reduced the burden of disease resulting from exposure to parasite infections. The consequences on naturally acquired immunity are unclear. A magnetic bead-based immunoassay (MBA) to assess antibody levels in populations living in endemic areas was previously evaluated. In this study, the effect of clinical attacks on immunity was analysed in three sentinel sites of Ivory Coast.

**Methods:**

Recombinant proteins or peptides derived from liver or blood stage antigens of *Plasmodiumfalciparum* (CSP, LSA1_41_, LSA3, SALSA, PF13-DBL1α_1_, GLURP, AMA1, MSP1p19, MSP4p20), the CSP of *Plasmodium malariae* and the salivary glands antigen of *Anopheles gambiae* (gSG6) were covalently linked to a colour-coded microsphere (Luminex™ beads) for the multiplex assay. ELISA was used for whole parasite extract antigen. Blood samples (n = 94) of patients consulting for symptomatic malaria attacks and living in three different malaria endemic settings (rural and periurban) were analysed.

**Results:**

Highly variable seroprevalence of antibody responses against parasite antigens was found ranging from 3 (gSG6) to 97 % (MSP4p20). A marked prevalence and significantly higher level of antibodies was found in patients from the rural site (Korhogo), those harbouring the lowest level of parasitaemia. The use of whole schizont extract could not discriminate immunity level, contrary to parasite-derived recombinant proteins or peptides. Prevalence of responders to LSA1_41_ and levels of antibodies to PF13 were significantly different between the three settings. Moreover, the post-treatment clearance of parasites was clearly associated with a significantly higher level of antibody response for almost 50 % of the parasite antigens tested.

**Conclusion:**

The multiplex MBA-Magpix technology assay provides an accurate high throughput monitoring of parasite-specific antibodies during symptomatic malaria. The levels of antibody responses may provide a risk criterion with respect to the degree of parasitic infection. Additionally, they can be used as an indicator in the implementation of malaria prevention and local control strategies.

## Background

Malaria is caused by a protozoan parasite of the genus *Plasmodium*. In Ivory Coast, malarial endemicity has been shown to be heterogeneous, depending upon various bio-ecological areas [1]. The spread and occurrence of malaria varies widely between villages and even within the same village. Malaria surveillance is monitored through a national malaria control programme (NMCP) and follow-up with relevant sentinel sites around the country. It has been established that areas of unstable, low, malaria transmission such Gambia and Kenya are characterized by a persistent risk of clinical malaria in older children and adults whereas in areas with stable, high-level of malaria transmission the risk of clinical malaria decreases markedly after the age of 5 years [2, 3].

In the recent years, the large-scale deployment of combined interventions strategies, including insecticide impregnated bed nets, rapid diagnostic tests and efficient combination therapy, led to a decrease of malaria burden in several sub-Saharan African areas [4]. Several tools are useful for follow-up progress of controls and decrease of malaria burden. For estimating the risk of malaria transmission or infection, the entomological inoculation rate (EIR) is considered as a gold standard. The EIR is estimated by the number of mosquito bites per man per night multiplied by the proportion of sporozoite positive mosquitoes [5]. Presently, with the decreasing transmission rate resulting from enlarged control programmes and scarcity of positive mosquitoes makes this method labour-intensive.

Another potential indicator of malaria transmission relies on ELISA-based serology investigations by measuring antibody responses against a set of antigens to evaluate exposure, such as mosquito salivary antigen [5–7], or different parasite-associated target antigens potentially associated with protection to malaria [8–10]. However, this technique can be used only for a limited number of antigens. Therefore, new strategy such as multiplex fluorescent bead-based assays have been developed by Luminex^®^ Corporation and allows the simultaneous testing of several serological markers together, providing capacity for accurate high throughput monitoring of malaria immunity [11–14].

Passive transfer experiments demonstrated that antibodies are major determinants of malarial protective immunity in human [15, 16]. Immunoglobulin G (IgG) antibody responses to a number of vaccine candidate antigens, including pre-erythrocytic antigens circumsporozoite protein (CSP) [17], liver-stage antigen 1 (LSA1) [17, 18], the blood-stage antigens as the merozoite surface protein 1 (MSP1) [19–21] and the Apical Membrane Antigen 1 (AMA1) [22]. These antigens have been associated with protection from clinical malaria in an area of stable transmission. Antibody responses to such vaccine candidates can also be investigated for use as indicators of past and recent malaria transmission. In the context of ongoing malaria elimination strategy, changes and variations of antibody responses in different settings could take place as useful monitoring tool requiring combination of different markers [10, 11, 19, 20, 23].

In the present study, malaria-related immunity was analysed by measuring antibody responses against 12 recombinant and peptide parasite antigens in individuals from Ivory Coast using a magnetic bead-based multiplex assay [24]. This pilot study involved an array of antigens previously investigated [11, 13] with the aim to provide immunology-related study in populations from this country. Recruitment involved three groups of approximately 30 patients on the day of consultation for clinical attacks in distinct geographical and malaria transmission settings.

Different profile and levels of antibody responses from malaria symptomatic patients according to different transmission settings were evidenced. These results indicate that the antibody response to antigens derived from *Plasmodium falciparum*, *Plasmodium malariae* or *Anopheles gambiae* can also be used as relevant biomarkers to evaluate follow-up and prevention measures at community level.

## Methods

### Study area, procedures for recruitment

Subjects were recruited in Korhogo, Man and Abobo, three Ivorian malaria-endemic areas included in the Sentinel National Network for Surveillance of Malaria. The protocol of surveillance was approved by the National Committee of the Ministry of Health. Individual informed written consent was obtained from participants/parents/guardians. In case of an illiterate patient, his/her thumb impression and signature of an independent witness were obtained. The study was conducted in accordance with the local laws and regulations, International Conference on Harmonization—Good Clinical Practice (ICH-GCP). The protocol was reviewed and approved by the Comité National d’Ethique et de Recherche de Côte d’Ivoire (N°56/MSLS/CNER-dkn).

Patients were enrolled in the pilot study in September–November 2013, after the rainy season i.e., after the peak of transmission.

Korhogo, located at 9°53′ latitude north and 6°49′ longitude west, bordering Mali and Burkina Faso, is a savannah area with a tropical climate and a transmission period of 6–8 months. The village of Man is located in the western forest and mountain area, at 7°24′ of latitude north, 7°33′ longitude west. Rainfall is abundant (1800 mm/year) and transmission occurs for 8–12 months. The site of Abobo is located in the southern part of the township of Abidjan, characterized by the presence of a lagoon with transmission occurring year round.

Recent data on the cumulative EIR in Korhogo [25] or Man were not available [26]. However, in the absence of exact EIR, morbidity data collected from health facility records in 2013, shown in Table 1, reflect the high level of transmission in these endemic areas.

**Table 1:** Context and characteristics of the study population

Characteristics	Abobo	Korhogo	Man
Geographical situation	Township Abidjan	Northern Savannah	Western forest
N° patients	31	32	31
N° children <5 years old	3	5	10
Age mean–median (range)	14–9.5 (2–54)	19.6–10.5 (1–70)	13.9–10 (1–59)
Mean parasitaemia and	103,706	28,474	52,336
[range parasitaemia] (in trophozoite per mL)	(3480–832,000)	(2030–92,800)	(247–465,800)
Haemoglobin: mean [range]–G/L	10.9 (8.1–12.9)	10.7 (6.3–16.1)	9.2 (6.0–12.5)
Thrombocytes: mean [range]–10^3^/μL	141 (32–309)	188 (37–530)	189 (26–536)
Duration of transmission season	All year	6–8 months	8–12 months
Individual use of LLINs^*^	27 %	10 %	20 %
Prevalence of clinical malaria^**^	5.2 %	14.7 %	18.3 %
% individuals with parasite clearance >24 h	90 %	13 %	32 %

^*^Long-lasting impregnated nets: individual inquiry for use of bed nets before consultation

^**^The mean national level of prevalence of clinical malaria in Ivory Coast is 10.57 % (2013)

This study involved 94 patients consulting for symptomatic fever in health centres: formation sanitaire Anonkoua-Kouté, centre de santé petit Paris, centre de santé urbain de ‘Libreville’, the respective healthcare centre in Abobo, Korhogo, Man. Patients were treated and followed up according to the standard national procedure. Diagnosis of malaria includes rapid diagnostic test (RDT), blood sampling for biological investigations and blood smear for parasite counting. Parasitaemia was counted on thick blood smears by two experienced microscopists. In case of discrepancy, smears were confirmed by a third counting. Characteristics of the three groups are summarized in Table 1. All patients were hospitalized, treated and followed up daily from day 0 to day 3 with artemether–lumefantrine combination. Parasitaemia was recorded every 24 h, up to two consecutive negative blood smears. Parasite clearance time (PCT) and its related clinical phenotype (delayed PCT) were recorded for each patient. These indicators are defined respectively as the time between treatment and the first negative slide, and as the proportion of patients still parasitaemic on days 2 or 3. An individual questionnaire for each patient recorded gravity symptoms (graded as none, moderate, intense), the use of bed nets and previous unprescribed individual use of anti-malarials. For all patients, almost complete PCT within 4 days was observed after treatment. The procedure included a recall of patients on days 7, 14, 21, 28, and 42 for confirmation of complete cure, tolerability and parasitological monitoring. No recrudescence of infections was recorded in any patients followed up. Plasma samples after biology processing at day 0 were collected upon centrifugation, and stored at −20 °C until further analysis.

### Antigens and peptides

Three soluble recombinant proteins and eight peptides conjugated to bovine serum albumin (BSA) specific to *P. falciparum*, *P. malariae* and *A. gambiae* salivary peptide gSG6 antigen (Ag) were included. BSA provided by peptide manufacturer was used as carrier control. The peptides used in this study were designed as already described [11]. A *N*-terminal cysteine residue was added to allow a unidirectional coupling to BSA and was done by the manufacturer (GenScript HK Inc.,Hong Kong, China,). Purity of each BSA-peptide was estimated >85 % by HPLC and mass spectrometry. The sequence of the peptides used was as follows:

- CSP: *(NANP)*_*9*_–*NVDPNVDPC*;
- LSA1_41_: *LAKEKLQEQQSDLEQERLAKEKLQEQQSDLEQERLAKEKEKLQC;*
- LSA3: *VLEESQVNDDIFNSLVKSVQQEQQHNVC;*
- GLURP: *EDKNEKGQHEIVEVEEILC*;
- SALSA: *SAEKKDEKEASEQGEESHKKENSQESAC;*
- AMA1: *YKDEIKKEIERESKRIKLNDNDDEGNKKIIAPRIFISDDKDSLKC;*
- *P. malariae* CSP: *(NAAG)*_*9*_–*NDAGC* and
- *A. gambiae* salivary peptide 1 (gSG6-P1): *EKVWVDRDNVYCGHLDCTRVATFC*.

The procedure for production and purification of the NTS–DBL1α1 domain of the PfEMP1 (*P. falciparum* Erythrocyte Membrane Protein-1) adhesin encoded by the 3D7/PF13 *var* gene has been reported elsewhere [27]. Soluble recombinant protein corresponding to *P. falciparum* MSP1p19 and MSP4p20 was produced in the baculovirus/insect cell expression system and purified by metallo-affinity chromatography as described [28].

### Coupling of antigen to beads

The covalent coupling of three recombinants antigens (PF13, PfMSP4p20 and PfMSP1p19) Ags and all the eight peptides to carboxylated magnetic Luminex microspheres by the carbodiimide reaction (Luminex Corp, Austin, USA) was done using the xMAP^®^Antibody Coupling Kit (ref 4050016, Luminex Corp, Austin, USA) according to the manufacturer’s instructions as already detailed by Perraut et al. (25). Briefly, 2.5 × 10^6^ beads from regions 26 to 39 were used in a working volume of 500 µL. All washing steps, buffer changing after 1–2 min centrifugation at 8000×*g*, magnetic pelletting with the Luminex^®^ Magnetic plate separator (Luminex Corp, Austin, USA), vortexing and sonication in water-bath sonicator to disperse the beads was done following manufacturer’s instructions. After carbodiimide hypochloride (EDC) activation step, 5 µg of Ag per million beads was added in the activation buffer and kept under rotation mixing in the dark for 2 h. After pelletting and washing, the supernatant was removed and replaced by 1 mL wash buffer and kept in the dark at 2–8 °C. Final count of remaining beads using cell counter showed a mean recovery of 98 % of the coupled beads. Efficient coupling of Ag was controlled using positive individual and pool of human sera. The coupled microspheres were kept in the washing/storage buffer at 4 °C in the dark until use.

### Bead-based assay for IgG antibodies

The custom-made Magnetic Bead-based MAGPIX^®^-Luminex Assay (MBA), performed in a dimly lit room, has been adapted to parallel the working steps used in the standard ELISA technique as previously described [24]. Plates included two positive controls: a pool of human Immune IgG (kind gift from Prof M Hommel) and a pool of 25 sera from clinically immune adults living in the village of Dielmo (a holo-endemic area of transmission in Senegal). Pools of European and African non-immune sera were included as negative control. Briefly, 2.5 µL aliquots containing 3000 beads per Ag from mix of microspheres, kept in an opaque vial, were dispensed to individual wells of a white, polystyrene, opaque, round-bottomed microtitre plate (Ref 103977741, Fisher Scientific, Illkirch, France). Duplicates of 100 μL plasma diluted 1:100 in PBS Tween 0.01 % BSA 1 % (PBSB) was added in wells, mixed and incubated with the beads protected from light on a microplate shaker (IKA^®^MTS, Wilmington, NC, USA) at 350 rpm for 45 min. After removal of plasma and two washing steps with 100 μL PBSB, 100 μL of phycoerythrin-labelled goat anti-human IgG diluted 1:500 in PBSB was added (gamma-chain specific, F(ab`)_2_ from Sigma, P-8047 St. Louis, MO, USA) and incubated in the dark with shaking at 350 rpm for 45 min. After two washes with 100 μL/well of PBSB, the beads were then resuspended in 120 μL PBSB and analysed on a Multiplex MAGPIX system (Millipore, USA) using the xPONENT 4.1 manufacturer’s software for acquisition. Antibody responses were expressed in median fluorescence intensity (MFI) per sample; individual positivity was considered when the signal was greater than [mean MFI signal +3 SD of 6 naïve control sera] as already described [12].

### ELISA procedure

IgG responses were quantified by ELISA in duplicate plasma samples diluted 1:100 as previously described using whole parasite extract Ag from schizonts 07/03 Dielmo strain adapted to culture [21, 29, 30]. The same positive and negative controls used for MBA were used in each assay as standards for plate comparability: pool of sera from adults living in the village of Dielmo, immune IgG (kind gift from Prof M Hommel) and pools of European and African non-immune sera. Results were expressed as OD ratio = OD sample/OD naive serum pool [30, 31]. Sera showing an OD ratio >2 were considered sero-positive, corresponding to the mean OD of naïve controls +2 SD.

### Statistical analysis

Antibody levels and prevalence of responders in different groups were compared using the Mann–Whitney signed rank test, the Spearman rank correlation test for non-normally distributed paired data and the Fisher exact test. In case of multiple associated comparisons, Bonferroni correction was applied. Statistical analyses were performed using Statview 5.0 (SAS Institute Inc. 2011. Base SAS^®^ 9.3 Procedures Guide. Cary, NC: SAS Institute Inc.) and R (R Core Team. R: a language and environment for statistical computing. R Foundation for statistical computing. Vienna, Austria. 2014).

To study the correlation between level of parasitaemia and levels of antibody responses, a log transformation was used. The log-transformed data were shown distributed according to normal law using Shapiro–Wilk test. So these variables were transformed into normally distributed variables before being included in the models. Generalized linear model was used to compare parasitaemia level (considered as dependent variable) to each clinical sign and setting (categorical variables) and antibody responses.

## Results

### Characteristics of the cohort

Recruitment was done after the rainy season for 30 patients per village, a limited proportion of children under 5 years old were included, except in Man (ten childrens vs five and three in Khorogo and Abobo, respectively). Mean age of the three groups was similar without any significant difference in the age distribution between the three settings (Table 1).

**Fig. 1:**
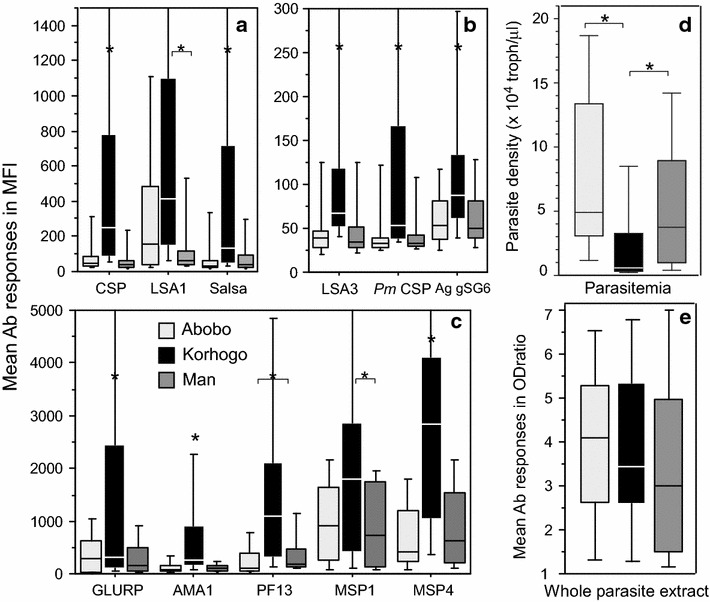
Mean levels of IgG responses to biomarkers and of parasitaemia in the three different sentinel sites. Antibody responses measured with multiplex magnetic bead based fluorescence assay are shown as box plot for median fluorescence levels (MFI) for the three settings, i.e., Abobo (*light grey*), Korhogo (*black*) and Man (*dark grey*). Antibody responses to pre-erythrocytic/erythrocytic antigens, *P. malariae* CSP and *A. gambiae* salivary peptide are shown in Part (**a**) and (**b**) (lower levels of MFI). In Part **c**, antibody responses to erythrocytic antigens are shown. Mean levels of parasitaemia and levels of antibody responses against whole schizont extract antigen are plotted in Part **d** and **e**, respectively. *Asterisks* or *asterisk with bracket* above bar charts indicate significant (P < 0.05) differences in levels of antibodies

As shown in Table 1 and Fig. 1, levels of parasitaemia measured upon recruitment were highly variable, with significantly lower levels in Korhogo, compared to the other settings (P < 10^−3^). These three settings are high risk for malaria, the prevalence of clinical malaria in Abobo is approximately 50 % lower than the mean national level and 150 % lower compared to the rural villages. Of note, urban patients recruited in Abobo suffered highly parasitic clinical episodes: 10 and 30 % of the patients harboured <10,000 or >100,000 trophozoites per μL, respectively, compared to 26 and 16 % in Man, and 56 and 0 % in Korhogo for these cut-off parasitaemia levels.

Importantly, treatment was highly efficient: 55, 42 and 3 % of patients overall cleared their parasitaemia in 24, 48 and 72 h, respectively. As detailed in Table 1, the most efficient clearance of parasites was observed in Korhogo followed by Man and then Abobo villages.

### Prevalence of antibody responses

Prevalence of antibody responses in the three settings against the 12 biomarkers is summarized in Table 2. Prevalence levels were highly variable, ranging from 3 % (IgG to gSG6 peptide) to 97 % (IgG to MSP4p20). For each setting, the overall mean level of prevalence increased from 37 % in Man, 42 % in Abobo, to 66 % in Korhogo.

**Table 2:** Levels and prevalence of IgG antibody responses in the different sentinel sites

Antibody responses to	Abobo			Korhogo			Man		
	IgG levels^f^	%	P^a^	IgG levels	%	P^b^	IgG levels	%	P^c^
Scz_Ag^e^	4.1 (1–7.3)	84	NS^d^	3.9 (1–10.4)	81	NS	3.6 (1–11)	68	NS
CSP	110 (16–920)	26	<10^−3^	602 (26–2834)	75	<10^−3^	133 (21–2139)	13	NS
LSA1_41_	376 (22–1475)	61	<0.05	1112 (31–8171)	84	<10^−3^	188 (27–1774)	32	<0.04
LSA3	70 (16–676)	10	NS	143 (40–1035)	31	NS	79 (19–637)	10	NS
SALSA	112 (18–886)	19	<10^−3^	785 (26–7656)	59	<0.004	74 (22–376)	23	NS
AMA1	145 (21–814)	39	<10^−3^	713 (47–3337)	84	<10^−3^	142 (37–747)	35	NS
GLURP	403 (20–2006)	45	NS	1485 (38–7701)	50	NS	344 (29–1440)	35	NS
MSP1p19	1031 (34–2834)	81	NS	2203 (77–9058)	88	NS	946 (42–3305)	68	NS
MSP4p20	817 (22–4231)	77	<0.03	3125 (90–10,674)	97	0.013	831 (49–2455)	74	NS
PF13	351 (21–3820)	45	<10^−3^	1887 (67–10,036)	91	NS	402 (33–1601)	71	NS
CSP *P. malariae*	60 (24–370)	10	<0.016	232 (33–3202)	38	<0.005	47 (25–267)	6	NS
gSG6 *An. gambiae*	66 (18–219)	6	NS	126 (25–452)	16	NS	83 (25–744)	3	NS

*P* Significant differences of prevalence by Fisher exact test

^a^Abobo *vs* Korhogo, ^b^ Korhogo *vs* Man, ^c ^Man *vs* Abobo, ^d^ *NS* non-significant, ^e^ ELISA measure of antibody responses against schizont extract expressed as mean OD ratio, ^f^ Mean [minimum–maximum] antibody responses expressed in OD ratio (schizont extract) and MFI

As detailed in Table 2, statistical comparisons of prevalence regarding individual biomarkers showed almost no significant differences between Man and Abobo except for LSA1_41_. In Korhogo, prevalence of antibody responses was significantly higher compared to the two other villages for CSP, LSA1_41_, SALSA, AMA1, MSP4p20, and *P. malariae* CSP. Prevalence of responders to PF13 was significantly lower in Abobo (45 %) compared to the two other settings (71 %, 91 % in Man and Korhogo, respectively).

### Levels of IgG antibodies responses in the three settings

Levels of antibody responses are summarized in Table 2, they are stated as Mean values and range [minimum–maximum] in ODratio and MFI. Comparison of levels of antibody responses are illustrated in Fig. 1 as box plot for all antigens (Fig. 1a, b, c) and whole parasite extract (Fig. 1e). As evidenced in Fig. 1, there are strong and selectively higher antibody responses against all biomarkers in individuals from the village of Korhogo, compared to the other settings (P < 10^−3^). When comparing responses to biomarkers in Abobo vs Man, no significant differences appeared except for PF13 (P = 0.04). Of note, the IgG response against whole parasite extract (from Senegalese strain 07/03) did not significantly discriminate any significant high level of antibody response in Korhogo as did the set of biomarkers.

### Inter-relation of antibody responses and correlation of antibody responses with age

Results of analysis for inter-relation between antibody responses against the different targets and the age of individuals are detailed in Table 3. The results calculated after Bonferroni correction for multiple comparisons was also included. There was no significant correlation between age and antibody responses against all targets except for GLURP peptide (rho = 0.5, P < 10^−3^). As summarized in Table 3, a strong interrelation between antibody responses to almost all target antigens was found, with an exception for somatic antigen and MSP1p19. Inter-relation between antibody responses was not significant for whole schizont extract antigen and MSP1p19; it was intermediate for LSA3 and GLURP peptides.

**Table 3:** Reciprocal inter-relation between age and IgG responses to the different biomarkers

Antigens	MSP4			CSP			LSA1			LSA3			GLURP			Salsa			MSP1			PF13			AMA1			PmCSP			Saliv			schz07/03		
	Rho	P	P_B_^*^	Rho	P	P_B_^*^	Rho	P	P_B_^*^	Rho	P	P_B_^*^	Rho	P	P_B_^*^	Rho	P	P_B_^*^	Rho	P	P_B_^*^	Rho	P	P_B_^*^	Rho	P	P_B_^*^	Rho	P	P_B_^*^	Rho	P	P_B_^*^	Rho	P	P_B_^*^
CSP	0.46	<10^−3^	Y																																	
LSA1	0.24	0.02	NS	0.49	<10^−3^	Y																														
LSA3	0.34	<10^−2^	NS	0.43	<10^−3^	Y	0.16	0.1	NS																											
GLURP	0.34	<10^−2^	NS	0.42	<10^−3^	Y	0.32	<10^−2^	NS	0.33	<10^−2^	NS																								
Salsa	0.44	<10^−3^	Y	0.63	<10^−3^	Y	0.28	<10^−2^	NS	0.43	<10^−3^	Y	0.48	<10^−3^	Y																					
MSP1	0.37	<10^−3^	Y	0.25	0.01	Ns	0.28	<10^−2^	NS	0.21	0.04	NS	0.26	0.01	NS	0.20	0.05	NS																		
PF13	0.44	<10^−3^	Y	0.39	<10^−3^	Y	0.21	0.04	NS	0.39	<10^−3^	Y	0.33	<10^−2^	NS	0.54	<10^−3^	Y	0.06	0.51	NS															
AMA1	0.46	<10^−3^	Y	0.52	<10^−3^	Y	0.18	0.07	NS	0.49	<10^−3^	Y	0.33	<10^−2^	NS	0.76	<10^−3^	Y	0.22	0.03	NS	0.54	<10^−3^	Y												
P m CSP	0.40	<10^−3^	Y	0.48	<10^−3^	Y	0.22	0.03	NS	0.54	<10^−3^	Y	0.37	<10^−3^	Y	0.55	<10^−3^	Y	0.0	0.1	NS	0.55	<10^−3^	Y	0.53	<10^−3^	Y									
Saliv	0.26	0.01	NS	0.32	<10^−2^	NS	0.04	0.64	NS	0.37	<10^−3^	Y	0.11	0.3	NS	0.39	<10^−3^	Y	0.29	<10^−2^	NS	0.41	<10^−3^	Y	0.46	<10^−3^	Y	0.30	<10^−2^	NS						
schz07/03	0.20	0.06	NS	0.20	0.04	NS	0.20	0.04	NS	0.06	0.5	NS	0.42	<10^−3^	Y	0.24	0.02	NS	0.25	0.01	NS	0.3	<10^−2^	NS	0.24	0.017	NS	0.14	0.17	NS	0.15	0.14	NS			
Age	0.21	0.04	NS	0.36	<10^−2^	NS	0.18	0.07	*NS*	0.15	0.14	*NS*	0.50	<10^−3^	Y	0.32	<10^−2^	NS	0.0	0.1	NS	0.3	<10^−2^	NS	0.20	0.05	NS	0.29	0.005	NS	0.03	0.73	NS	0.30	<10^−2^	NS

* Probablity of correlation after bonferoni correction for all determinations (n = 78; *P* < 0.0006), Y significant, *NS* non significant

In this double entry table are summarized results from reciprocal analysis of correlation between antibody responses to all antigens, and between age and antibody responses on the last line. Strong inter-relation between IgG responses against all antigens was evidenced (55 were significant out of 66, P < 0.05), remaining significant for 30 out of 66 after Bonferroni correction (P < 0.0006). Age correlated with antibody responses for 7/12 antigens remaining significant only for GLURP antigen after Bonferroni correction

### Relationship of antibody responses with circulating parasitaemia, haematological measures and clinical criteria

There was no correlation between parasitaemia observed at recruitment (or log-transformed parasite density) with antibody responses against all antigens except a significant negative correlation with IgG responses to LSA3 (P < 10^−3^) rho = −0.35 and PF13 (P < 10^−4^) rho = −0.41.

When stratifying parasite densities under and over 50,000 trophozoites per μL, only antibody responses to PF13 remained significantly lower (P = 0.01) in individuals with high levels of parasites (approximately one-third of the overall cohort). A significant relationship was found between parasitaemia and thrombocytaemia (P < 0.001), thrombocytaemia decreased when parasitaemia increased.

**Fig. 2:**
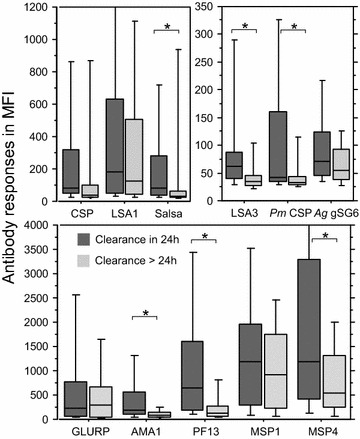
Distribution of IgG responses against biomarkers as function of delay for parasite clearance after treatment. Dichotomization of antibody responses expressed as mean median fluorescence levels (MFI) are plotted as box plot for the overall cohort of patients as function of either early clearance (24 h, *dark grey*) or later clearance (48 and 72 h, *light grey*). *Asterisks* indicate a significant (P < 0.05) different level of antibody level

The relationship between immune responses and the efficiency of treatment was analysed by stratifying clearance of parasitaemia between early (<24 h) and later (48–72 h) delay of blood smear negativation. As shown in Fig. 2, there was a general trend for higher antibody levels in individuals with rapid clearance. In this group, higher levels were found significant for 50 % of the biomarkers, i.e., LSA3, SALSA, AMA1, PF13, MSP4p20, and *Pm*CSP.

When investigating correlates with the records of clinical signs, a significant relationship was found only between parasitaemia and nausea (P < 0.01), parasitaemia was higher in patients with nausea. In the multivariate analysis, studying the relationship of parasitaemia with PF13, LSA3, thrombocytaemia, nausea, village, the last model was based only on PF13 and thrombocytaemia. Parasitaemia decreased with PF13 level and increased when thrombocytaemia decreased.

## Discussion

The present study was undertaken to draw a first profile of antibody responses to a large array of antigens in three sentinel settings for malaria national surveillance and treatment in Ivory Coast, a country with a high level of endemicity [4]. There are few large-scale studies analysing precisely the prevalence of *P. falciparum* malaria and its recent history in Ivory Coast. However, observed *P. falciparum* prevalence in previous surveys from central and south-central Ivory Coast [1, 32, 33] underlined that malaria is highly endemic and characterized by considerable heterogeneity. In addition, the complex local social-ecological contexts, including population influx, might play a role resulting in an increase of presumed and reported clinical cases from 2.8 to 4.7 million in 2012 and 2013, respectively [4]. Transmission can also change depending upon climate and rainfall [34], as well as potential changes of vector behaviour, such as adaptation of *A. gambiae* to polluted water [35] or the strong natural heterogeneity in entomologic parameters of malaria transmission [36].

In this context, affordable tools for evaluation and monitoring of malaria control interventions, such as immune biomarkers, can play a valuable role [9–11, 14]. Indeed, individual risk of infective bites depends substantially of the use of bed nets, and here the proportion of use was declared <30 % and slightly different between the settings Abobo > Man > Korhogo (Table 1). In addition, deep change in behaviour of *A. gambiae* sub-population can add a possible source of increasing transmission [37].

Recruitment of patients with non-severe clinical malaria was a first pilot approach, regarding which clinical infection by *P. falciparum* strongly stimulates immune responses against all components of the parasite. Thus, all patients had a comparable, individual, clinical susceptibility to infection that necessitated consultation and hospitalization, independently of the setting and the individual natural history of infection. There was a clear and highly significant, unexpected, difference of antibody responses between one of the setting (Korhogo) and the other two.

Most sero-epidemiology studies rely on cross-sectional or longitudinal follow-up of individuals before or after clinical episodes, including prospective analysis to establish relationship between antibody responses against one antigen [17–19, 21–23, 38] or several antigens and protection against clinical malaria [20, 39]. Excepting individuals with active asymptomatic parasite carriage, almost all studies involve a relatively stable state in host-parasite interaction, an approach in which serological analyses are potentially relevant indicators of malaria transmission [10, 40]. For example, a single antigen AMA1 has been used for mapping effectiveness of control intervention [41]; in another case, several antigens were used as markers of exposure and malaria risk [39]. A similar methodology was undertaken in this study, underlining a clear significant higher immunological background in Korhogo. In this site, transmission reflected by prevalence of clinical malaria, was high but slightly lower than in Man. Here, as the recruitment involved symptomatic cases, antibodies measured against targeted antigens may play no causal role in protection but rather reflect the individual boosting capacity of immune responses. A higher level of antibody responses against the set of antigens was found in individuals with significantly stronger immune background but still susceptible to parasite invasion with clinical attack. This could explain the absence of relationship between parasitaemia and antibody responses observed, despite significant inter-relation between antibody responses.

Interestingly, the whole parasite extract from 07/03 in vitro-adapted strain from Dielmo has been already used as a relevant marker for follow-up of immunity levels [29, 42, 43], but did not correlate with clinical protection in prospective studies [21]. In Ivory Coast, levels and prevalence of antibody responses to the whole parasite extract did not show any difference between the three settings, contrary to defined antigens in Korhogo. Such discrepancy could be related to some substantial difference between this particular Senegalese strain and the circulating strains in Ivory Coast. This observation has to be further confirmed by testing a reference strain and/or a locally adapted strain.

One main question raised by the measure of multiplex reactogenicity profile on day of consultation is the search for potential immune marker(s) able to predict a differential susceptibility to the observed level of infection. Only two antigens induced antibody responses significantly different between the sites of Abobo and Man: the incidence of responders to LSA1_41_ and the levels of antibody responses to PF13. PfEMP1–PF13 antigen was shown as a relevant biomarker of cumulative acquisition of immunity in endemic setting [27, 44] and appeared significantly related to parasite levels. Such capacity has to be further confirmed with larger cohorts from more settings.

Susceptibility to infection can be reflected by the level of parasitaemia and by the degree of individual severity of clinical signs. The level of parasitaemia was significantly lower in Korhogo compared to the two other sites and there was a clear decreasing gradient of high level parasitaemia from Abobo (30 %) to Man (16 %) and Korhogo (0 %). The multivariate analysis investigating correlation between level of parasitaemia, according to each clinical signs and setting, underlined only nausea as a significant clinical sign. This is in line with observation that severity of clinical outcome in a mild malaria episode is not related to the level of parasitaemia or acquired immunity, once susceptible [45]. However, despite substantial difference in the magnitude of parasite invasion according to the setting, treatment was 100 % efficient by 72 h. Within the period covered by this study, there was no indication of delayed response to artemisinin combination therapy (ACT). Parasites were cleared rapidly, leaving very few patients with a low parasitaemia on day 3: only 1.5 % of the patients were still positive on day 3 which is under the threshold of 3 % indicating delayed response [46].

Importantly, this study shows clearly the potential impact of host immunity on malaria treatment outcome. A fast clearance of circulating parasite (24 h) was associated with higher level of immunity. This general trend, shown in Fig. 2, was significant for 50 % of the biomarkers. These results are in agreement with several studies involving sulfadoxine–pyrimethamine or amodiaquine treatment, showing a significant association of higher levels of antibodies to GLURP [47], AMA1 [48] and MSP1 [49] antigens with a lower risk of treatment failure. Another study showed the contribution of antibodies to the Ring Erythrocyte Surface antigen to the therapeutic response in Thai patients treated with artesunate [50]. Indeed, the positive contribution of host immunity for rapid parasite clearance underlined in this study is outlined by multi-site recruitment testing, contrary to previous investigations [47–50]. The use of antibody responses to biomarkers underlined clearly the site of Korhogo as different from the other two; parasite transmission seems to remain high, marked by higher boosting capacity of antibody responses. Such observation could possibly be related to lower application of individual prevention measures. This was confirmed by a two–three-times lower utilization of bed nets, as recorded by individual questionnaire, and the differential impact on antibody responses to gSG6 salivary peptide. However, such individual behaviour has to be further confirmed. Serological tools, such as *Anopheles* salivary peptide gSG6-P1 antigen, have been shown to be a new relevant marker of risk for malaria transmission [5–7, 51–53]. A significant higher level of IgG against the salivary peptide in Korhogo compared to other villages was found. However, seroprevalence did not significantly differ and MFI levels were low compared to other biomarkers. This lower sensitivity may be related to the intrinsic multiplex technique compared to ELISA, based on a high concentration of peptide coating (20 µg/Ml) and lower serum dilution for detection (1:20 instead of 1:100 in MBA) [5]. Further analysis using this biomarker may rather require ELISA than MBA for enhanced sensitivity.

Accurate analysis of immune responses from malaria symptomatic individuals can underline heterogeneity in individual degree of exposure and bring important information on the effective application of control measures at a community level.

## Conclusion

This pilot study suggests that measures of antigen reactivity profiles using the MBA can conveniently contribute to follow-up and monitoring of integrated malaria control measures. A panel of antigens, covering targets from different parasite stages is preferentially required, including antigens from other *Plasmodium* species. However, further investigations are required, including antigen targets from other species, enlarged to multiple sites and comprising enrolment of young individuals. Immunity profiling using biomarkers has the potential for immunological mapping of intervention and help with prevention measures towards malaria elimination.
